# Comparative efficacy of treatments for patients with knee osteoarthritis: a network meta-analysis

**DOI:** 10.1186/s40001-020-00426-1

**Published:** 2020-07-20

**Authors:** Bingtong Li, Yuzheng Zhang, Liqi Bi

**Affiliations:** 1 Department of Rheumatology and Immunology, China-Japan Union Hospital of Jilin University, No. 126 Xiantai Street, Changchun, 130000 Jilin China; 2 Department of Epidemiology and Biostatistics, School of Public Health, Jilin University, Changchun, 130021 Jilin China

**Keywords:** Knee osteoarthritis, Hyaluronic acid, Corticosteroids, Platelet-rich plasma, Efficacy, Network meta-analysis

## Abstract

**Background:**

Knee osteoarthritis is a common cause of musculoskeletal pain and a leading cause of disability and healthcare economic burden. The optimum treatment for knee osteoarthritis is still inconclusive. A network meta-analysis is required to assess the efficacy and safety of treatments and provide more scientific medical evidence.

**Methods:**

Relevant studies were searched through PubMed, Embase, and Cochrane Library electronic databases from the inception to October 2018. Continuous outcomes such as pain, stiffness, physical function and total scores were expressed as the mean differences with 95% credible interval. Surface under the cumulative ranking curve illustrated the rank probability of each therapy under different outcomes.

**Results:**

Nineteen studies were included in this study, with a total of 2395 patients. For knee pain, platelet-rich plasma (0.691) was ranked at the first place, followed by hyaluronic acid combined with platelet-rich plasma (0.670) and hyaluronic acid (0.402). In terms of stiffness, hyaluronic acid combined with platelet-rich plasma (0.743) enjoyed the highest value, platelet-rich plasma (0.603) was the next and hyaluronic acid (0.386) was the third. As for physical function, the rank was hyaluronic acid combined with platelet-rich plasma (0.772), platelet-rich plasma (0.608) and hyaluronic acid (0.343). For total scores, the order given by surface under the cumulative ranking was hyaluronic acid combined with platelet-rich plasma (0.765), platelet-rich plasma (0.624) and hyaluronic acid (0.37).

**Conclusions:**

Hyaluronic acid combined with platelet-rich plasma showed the best efficacy in improving stiffness, physical function, and total scores, while platelet-rich plasma appeared the best in terms of pain reduction.

## Background

Knee osteoarthritis (OA) is a degenerative disease with clinical manifestations, including joint pain, tenderness, joint swelling restricted movement as well as joint deformities and a leading cause of disability and healthcare economic burden [1, 2]. More than 50% aged 60–75 years old people owned radiological evidence of knee OA and 80% of population over 75 years old suffered from knee OA [3, 4]. In recent years, the incidence of OA has increased and presents as a serious threat to human health and quality of life [5]. The current optional treatments for knee OA include oral anti-inflammatory drugs, physical therapy, topical anti-inflammatory gels and intra-articular injections [6]. The intra-articular injection consists of various drugs such as corticosteroid, hyaluronic acid (HA) and platelet-rich plasma (PRP).

Corticosteroids are commonly utilized as intra-articular injection agents [7]. Significant effects of corticosteroids have been reported by several studies for treating the knee OA [8–10]. The short-term benefits of intra-articular corticosteroid injection are well recognized, while the long-term benefits and the value of repetitive injections are still debatable [11, 12].

OA is characterized by degenerative of articular cartilage, osteophyte formation, changes in the synovial membrane, subchondral bone sclerosis, and reduced viscosity of synovial fluid [13]. HA is a widely distributed, liner glycosaminoglycan constituent of cartilage, synovial fluid, skin and aqueous humor [14]. It has been proved that synovial fluid from arthritic joints contains lower concentrations of HA than that from normal joint [15]. Since the elasticity and viscosity of synovial fluid are directly proportional to HA content and integrity, intra-articular injection of HA is a rational approach to the treatment of OA [14]. Although beneficial effects on pain, function and patient global assessment have been documented, the real entity of improvement and which of the many available HA products can offer the best results is not clear [16].

Platelet-rich plasma (PRP) has also been introduced as an alternative treatment for patients with knee OA [17]. PRP comprised a potent cellular milieu containing platelet concentrations above baseline, as well as an undifferentiated mixture of anti-inflammatory, pro-inflammatory, anabolic and catabolic mediators in an attempt to stimulate a supra-physiologic response and elicit the body’s natural healing potential [17–19]. The analysis of Zhang’s et al. review demonstrated that published literatures supported the efficacy of PRP in the treatment of tendinous, cartilaginous, ligamentous, and muscular injuries [12]. In a systematic review and meta-analysis, Laudy et al. found that patients treated with PRP injections reduced more pain than those receiving placebo injections [20]. Nevertheless, the level of evidence was limited since the risk of bias was high. Although intra-articular saline injections are used commonly as placebos in many trails, there has been increasing recognition that the application of intra-articular saline may have some effect [21, 22]. However, this result was drawn from clinically heterogeneous samples [22].

Controversies remain regarding in the treatment of knee OA. Many articles compared HA and PRP to determine which therapy was better in terms of pain reduction. In 2016, a meta-analysis reported that PRP was more effective when compared with HA [23]. Cole et al. observed that there was no difference between HA and PRP in the primary outcome in terms of the patient-reported Western Ontario and McMaster Universities Osteoarthritis Index (WOMAC) pain score [6]. Similarly, studies of HA and corticosteroid injection in patients with knee OA have displayed various results [24, 25].

Therefore, a network meta-analysis (NMA) is required due to contradictions and to give more scientific evidence for the choice of treatments. We performed the first NMA to evaluate the efficacy and safety of five different treatments for patients with OA, including corticosteroid, HA, PRP, placebo and HA + PRP, in regard to their performance on pain, stiffness, physical function or total assessed with quantitative scores by WOMAC, in order to provide optimal treatments for knee OA patients.

## Methods

### Literature search

Relevant studies were searched through PubMed, Embase, and Cochrane Library electronic databases from the inception to October 2018. All searches were limited to randomized controlled trials (RCTs) in humans. These following search terms and their corresponding synonyms were applied for systematic searching: “knee osteoarthritis”, “platelet-rich plasma”, “hyaluronic acid”, “corticosteroid”, “placebo”, and “randomized controlled trial”. There were no limits on language and publication date. Two authors independently retrieved potential eligible articles.

### Study selection

Criteria for eligibility of published studies were as follows: (1) study design should be double/triple-blinded RCTs; (2) follow-up period should be at least 1 month; (3) study subjects should be patients diagnosed with knee OA according to the American College of Rheumatology criteria [26], Ahlbäck’s criteria [27], or by the radiological assessment of Kellgren and Lawrence [28]; (4) studies contained at least two of the following treatments: corticosteroid, HA, PRP and HA + PRP; (5) outcomes included pain, stiffness, physical function or total assessed with quantitative scores by WOMAC.

The exclusion criteria included the following items: (1) patients receiving previous surgery or joint arthroplasties of the investigational knee; (2) studies with insufficient data, such as the article has only one study group or has not the main outcomes; (3) duplicated patients or studies; (4) systematic reviews, conference reports or comments; (5) cell or animal experiments.

### Data extraction

The data were independently extracted by two reviewers. The following information was extracted from the included articles: first author’s name, publication year, country, blinding, treatments of each groups, samples size, the degree of disease, the number of injection, duration of follow-up, age, sex ratio and BMI. Any disagreement concerning the data extraction was resolved by discussion. The outcomes included WOMAC pain, stiffness, physical function and total scores. WOMAC is a 24-item questionnaire, which is divided into three subscales measuring pain, stiffness and physical function [29]. Pain contains 5 items and score ranges from 0 to 20. Stiffness contains 2 items and score ranges from 0 to 8. As for physical function, it includes 17 items and score ranges from 0 to 68 [29].

### Statistical analysis

All data analysis was performed through a Bayesian model in WinBUGS (MRC Bio-statistics Unit, Cambridge, UK). Endnote software (version X8.0) was utilized to manage articles. Continuous outcomes such as pain, stiffness, physical function and total scores were expressed as the mean differences (MD) with 95% credible interval (CrI). A significant difference could be found by 95% CrI which did not contain 0 for MD. The net plot was used to show the connection between each treatment for four outcomes. The result of NMA was indicated by the slash table. Moreover, the comparison between direct and indirect data was demonstrated by the node-splitting table. Surface under the cumulative ranking curve (SUCRA) illustrated the rank probability of each therapy under different outcomes. The larger SUCRA value stood for the better rank of the intervention. Finally, the funnel plot was generated to assess the publication bias.

## Results

### Included studies

**Fig. 1:**
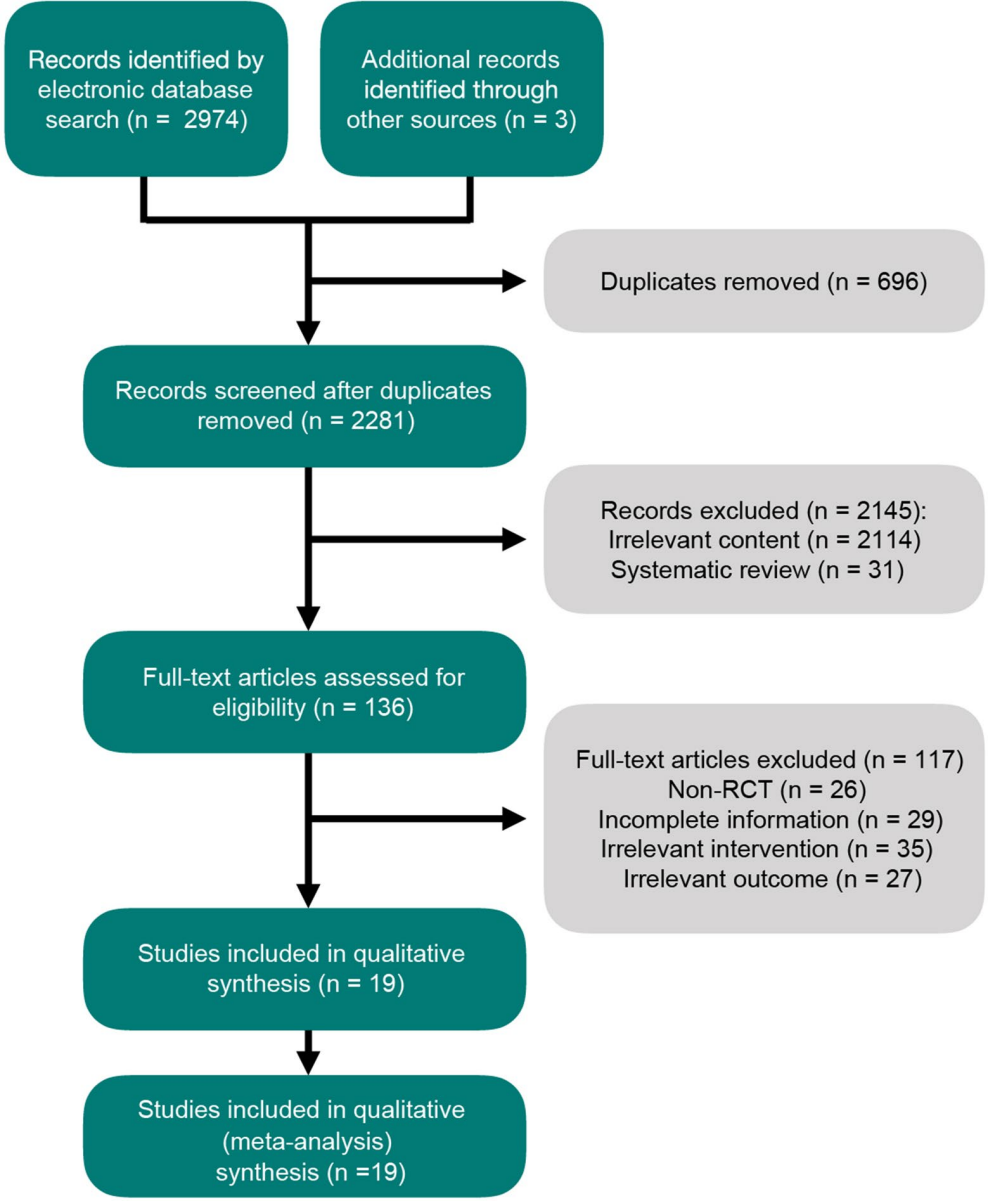
Flow diagram summarizing results of study identification and selection

As shown in the flowchart (Fig. 1), 2974 literatures were searched from electronic database. After adding 3 studies from the citations of related review, 2977 studies were assessed for the next step. Then 696 studies were removed due to duplicates, leaving 2281 literatures assessed to be eligible. By analyzing the titles and abstracts, 2145 articles were excluded for irrelevant content (2114) and systematic review (31). No non-English article was included due to not being eligible for the inclusion criteria after analyzing the abstract. By reading the full articles, a total of 117 studies were excluded for not randomized controlled trial (26), insufficient data (29), low relevance (35) and unavailability of specific data (27). Ultimately, 19 double-blinded studies were involved in our analysis.

### Study characteristics

**Fig. 2:**
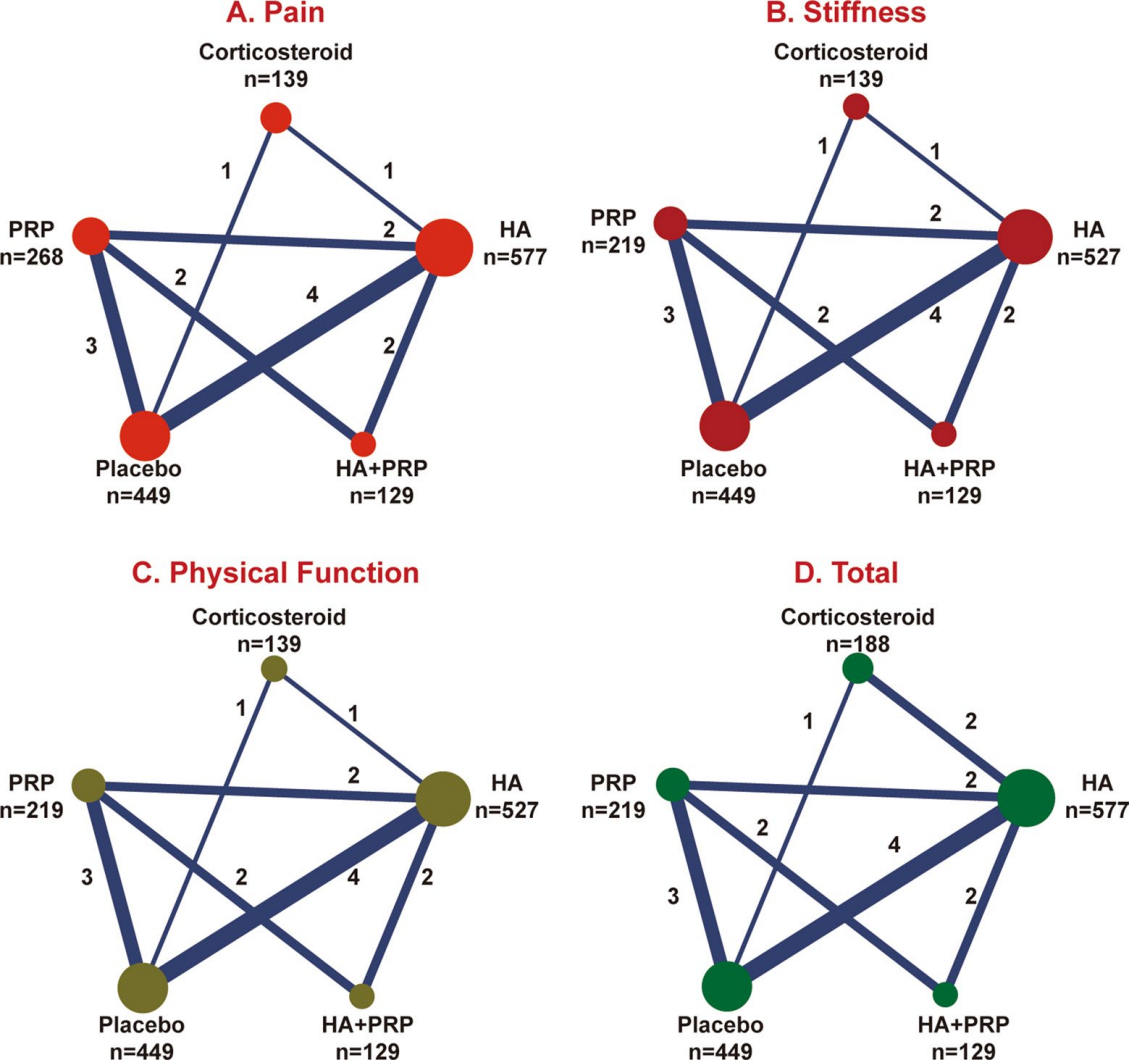
Net plots of four outcomes: pain, stiffness, physical function and total (sum of the other 3 outcomes). Line width is proportional to the number of trials comparing treatments with numbers on the lines representing the exact number. Circle area represents the cumulative number of patients in each intervention. *HA* hyaluronic acid, *PRP* platelet-rich plasma

All included studies were RCTs published between 2002 and 2018, with a total of 2395 patients. The sample size varied between 10 and 174 and the average age varied between 46.2 and 69.6 years old. Follow-up period ranged from 8 weeks to 2 years. The mean number of female ratio ranged from 27.3% to 100% of patients. The characteristics of 19 included studies are presented in Table 1. As for five treatments, the comparison between HA and placebo was the most frequently reported one and the pair of PRP and placebo was the second. In these 19 studies, 17 were two-arms, while the other 2 were three/four-arms, respectively. The number of injection and dosage of each treatment has also been presented. Figure 2 shows more details on direct comparison of different therapies.

**Table 1:** Basic characteristics of included RCTs

Author, year, country	Disease degree	Treatments	Size	Age	Male (%)	BMI	Dosage	No. of injection	Follow-up
Yu, 2018, China	KL grade I–IV	PRP	104	46.2	48.10		8 ml PRP	5	52 weeks
		HA	88	51.5	54.50		0.2 mg HA	5	52 weeks
		PRP + HA	96	46.5	52.10		8 ml PRP + 0.2 mg HA	5	52 weeks
		Placebo	72	56.2	58.30		NM	5	52 weeks
Rahimzadeh, 2018, Iran	KL grade I–II	PRP	21	65.5	47.60		7 ml PRP	1	26 weeks
		Placebo	21	64.3	52.40	28.6	7 ml 25% dextrose	1	26 weeks
McAlindon, 2017, American	ACR criteria; KL grade II–III	Corticosteroid	70	59.1	47.10	28.3	4 mg corticosteroid	8	2 years
		Placebo	70	57.2	45.70	30.8	1 ml saline	8	2 years
Tammachote, 2016, Thailand	ACR criteria	HA	50	62.6	14.00	31.7	6 ml HA	1	26 weeks
		Corticosteroid	49	61	26.00		40 mg TA	1	26 weeks
Cole, 2016, American	KL grade I–III	PRP	49	55.9	56.00		4 ml PRP	3	52 weeks
		HA	50	56.8	60.00	27.4	16 mg	3	52 weeks
Paterson, 2016, Australia	ACR criteria; KL grade II–III	PRP	11	49.9	72.73	29	3 ml PRP	1	12 weeks
		HA	10	52.7	70.00	27.9	3 ml HA	1	12 weeks
Lana, 2016, American	KL grade I–III	HA	36	60	91.70	30.9	20 mg HA	3	52 weeks
		PRP	36	60.9	80.60	28.2	5 ml PRP	3	52 weeks
		PRP + HA	33	62	81.80	27.4	20 mg HA + 5 ml PRP	3	52 weeks
Askari, 2016, Iran	KL grade II–III	Corticosteroid	69		17.40	29.2	2 cc HA	1	13 weeks
		HA	71		12.70		40 mg corticosteroid	1	13 weeks
Forogh, 2016, Iran	KL grade II-III	PRP	24	59.1	29.20		5 ml PRP	1	26 weeks
		Corticosteroid	24	61.1	37.50	75.8	40 mg corticosteroid	1	26 weeks
Filardo, 2015, American	KL grade I–III	PRP	94	53.3	63.80	75.8	5 ml PRP	3	52 weeks
		HA	89	57.6	58.40	26.6	2 ml HA	3	52 weeks
Rayegani, 2014, Iran	ACR criteria; KL grade I–IV	PRP	31	58.1	6.50	26.9	4–6 ml PRP	2	26 weeks
		Placebo	31	54.7	6.50	28.2	NM	NM	26 weeks
Patel, 2013, India	ACR criteria; Ahlbäck grade I–II	PRP	27	53.1	40.70	27.3	8 ml PRP	1	26 weeks
		Placebo	23	53.7	26.00	26.3	8 ml saline	1	26 weeks
Filardo, 2012, Italy	KL grade I–III	PRP	54	55	68.50	26.2	5 ml PRP	3	52 weeks
		HA	55	58	56.40	27	NM	3	52 weeks
Huang, 2011, China	ACR criteria; KL grade II––III	HA	100	65.9	26.00	26	20 mg HA	5	25 weeks
		Placebo	100	64.2	22.00	25.7	2 ml saline	5	25 weeks
Diracoglu, 2009, Turkey	ACR criteria; KL grade II–III	HA	40			25.4	NM	1	26 weeks
		Placebo	20				NM	1	26 weeks
Lundsgaard, 2008, Denmark	KL grade I–IV	HA	84	68.8	42.9		2 ml HA	4	52 weeks
		Placebo	84	69.6	47.60	29.6	2 ml saline	4	52 weeks
Ubuku, 2004, Turkey	ACR criteria	HA	20	52.6	30.00	29.3	2 ml HA	1	8 weeks
		Placebo	10	57.6	0.00		2 ml saline	1	8 weeks
Altman, 2004, American	ACR criteria	HA	172	62.9	54.00		60 mg HA	1	26 weeks
		Placebo	174	63.3	36.00		3 ml 0.9% saline	1	26 weeks
Karlsson, 2002, Sweden	Ahlbäck grade I–II	HA	76		35.00		4.5 ml HA	3	52 weeks
		Placebo	57		39.00		3 ml saline	3	52 weeks

*HA* hyaluronic acid, *PRP* platelet-rich plasma, *ACR* American College of Rheumatology, *KL* Kellgren and Lawrence, *NM* not mentioned, *TA* triamcinolone acetonide

### Efficacy and safety results

**Fig. 3:**
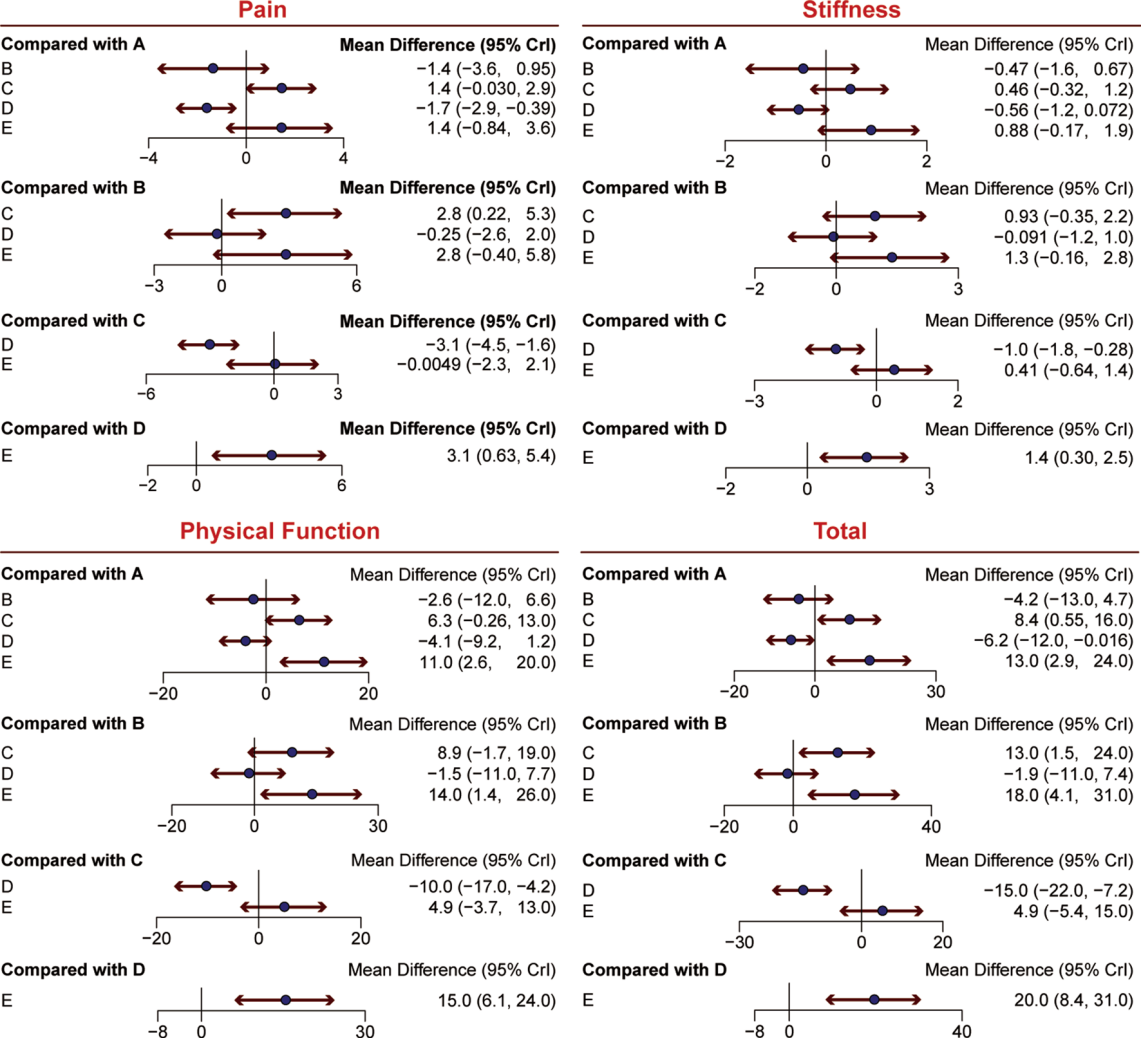
Forest plots of four outcomes: pain, stiffness, physical function and total (sum of the other 3 outcomes). The mean difference with 95% credible intervals (CrIs) indicates relative efficacy of treatment compared to another treatment. *A* hyaluronic acid, *B* triamcinolone, *C* platelet-rich plasma, *D* saline, *E* hyaluronic acid + platelet-rich plasma

For knee pain, better improvement can be found in patients receiving PRP treatment compared with those receiving corticosteroid [PRP vs. Corticosteroid: MD = 2.8, 95% CrI (0.22, 5.3)]. Compared with placebo, there was statistical significance in the improvement of KOA in patients treated with HA [Placebo vs. HA: MD = − 1.7, 95% CrI (− 2.9, − 0.39)], PRP [Placebo vs. PRP: MD = − 3.1, 95% CrI (− 4.5, − 1.6)] and HA + PRP [HA + PRP vs. Placebo: MD = 3.1, 95% CrI (0.63, 5.4)]. These results are shown in Fig. 3 and Table 2. Similarly to the SUCRA value in Table 3, PRP (0.691) was ranked at the first place, followed by HA + PRP (0.670) and HA (0.402).

**Table 2:** Network meta-analysis results of efficacy outcomes

Pain					
HA	− 0.47 (− 1.6, 0.67)	0.46 (− 0.32, 1.25)	− 0.56 (− 1.18, 0.07)	0.88 (− 0.17, 1.86)	Stiffness
1.4 (− 0.95, 3.65)	Corticosteroid	0.93 (− 0.35, 2.22)	− 0.09 (− 1.19, 1.02)	1.35 (− 0.16, 2.78)	
− 1.41 (-2.9, 0.03)	− 2.82 (− 5.35, − 0.22)	PRP	− 1.02 (− 1.76, − 0.28)	0.41 (− 0.64, 1.39)	
1.66 (0.39, 2.89)	0.25 (− 1.99, 2.56)	3.07 (1.62, 4.53)	Placebo	1.44 (0.3, 2.49)	
− 1.4 (-3.56, 0.84)	− 2.81 (− 5.82, 0.4)	0 (− 2.11, 2.25)	− 3.06 (− 5.36, − 0.63)	HA + PRP	
Physical function					
HA	− 4.25 (− 12.88, 4.66)	8.37 (0.55, 16.49)	− 6.17 (− 12.15, − 0.02)	13.32 (2.86, 23.91)	Total
2.62 (− 6.64, 11.74)	Corticosteroid	12.62 (1.48, 23.72)	− 1.94 (− 11.39, 7.41)	17.56 (4.07, 30.85)	
− 6.3 (− 12.98, 0.26)	− 8.92 (-19.39, 1.74)	PRP	− 14.56 (− 22.02, − 7.19)	4.94 (− 5.43, 15.2)	
4.14 (− 1.19, 9.21)	1.51 (-7.66, 10.7)	10.43 (4.21, 16.58)	Placebo	19.5 (8.4, 30.51)	
− 11.15 (− 19.83, − 2.62)	− 13.78 (− 26.09, − 1.39)	− 4.85 (− 13.35, 3.65)	− 15.3 (− 24.42, − 6.06)	HA + PRP	

*HA* hyaluronic acid, *PRP* platelet-rich plasma

**Table 3:** The SUCRA probabilities of all treatments on clinical outcomes

Treatment	Pain	Stiffness	Physical function	Total
HA	0.402	0.386	0.343	0.37
Corticosteroid	0.152	0.172	0.195	0.17
PRP	0.691	0.603	0.608	0.624
Placebo	0.084	0.096	0.083	0.07
HA + PRP	0.67	0.743	0.772	0.765

*HA* hyaluronic acid, *PRP* platelet-rich plasma

In terms of stiffness, HA + PRP and PRP demonstrated its significant advantages over placebo [HA + PRP vs. Placebo: MD = 1.4, 95% CrI (0.30, 2.5), Placebo vs. PRP: MD = − 1.0, 95% CrI (− 1.8, − 0.28)]. No remarkable improvement was observed in other comparisons, as displayed in Table 2 and Fig. 3. Illustrated by the results of SUCRA in Table 3, HA + PRP (0.743) enjoyed the highest SUCRA value, PRP (0.603) was the next and HA (0.386) was the third.

As for physical function, HA + PRP exhibited a better effect than HA, corticosteroid and placebo with significant difference [(HA + PRP vs. HA: MD = 11.0, 95% CrI (2.6, 20.0), HA + PRP vs. Corticosteroid: MD = 14.0, 95% CrI (1.4, 26.0), HA + PRP vs. Placebo: MD = 15.0, 95% CrI (6.1, 24.0)]. Besides, PRP had a better efficacy than placebo in the treatment of KOA [Placebo vs. PRP: MD = − 10.0, 95% CrI (− 17.0, − 4.2)]. This was also verified by SUCRA values in Table 3. The rank was HA + PRP (0.772), PRP (0.608) and HA (0.343).

For total scores, HA + PRP performed better than corticosteroid, HA and placebo with statistical significance [HA + PRP vs. Corticosteroid: MD = 18.0, 95% CrI (4.1, 31.0), HA + PRP vs. HA: MD = 13.0, 95% CrI (2.9, 24.0), HA + PRP vs. Placebo: MD = 20.0, 95% CrI (8.4, 31.0)]. In addition, PRP functioned better than corticosteroid, HA and placebo with significant difference [PRP vs. Corticosteroid: MD = 13.0, 95% CrI (1.5, 24.0), PRP vs. HA: MD = 8.4, 95% CrI (0.55, 16.0), Placebo vs. PRP: MD = − 15.0, 95% CrI: (− 22.0, − 7.2)]. Moreover, HA was better than placebo for patients with KOA [Placebo vs. HA: MD = − 6.2, 95% CrI (− 12.0, − 0.016)]. The order given by SUCRA in Table 3 was HA + PRP (0.765), PRP (0.624) and HA (0.37).

### Inconsistency assessment and risk of bias

**Fig. 4:**
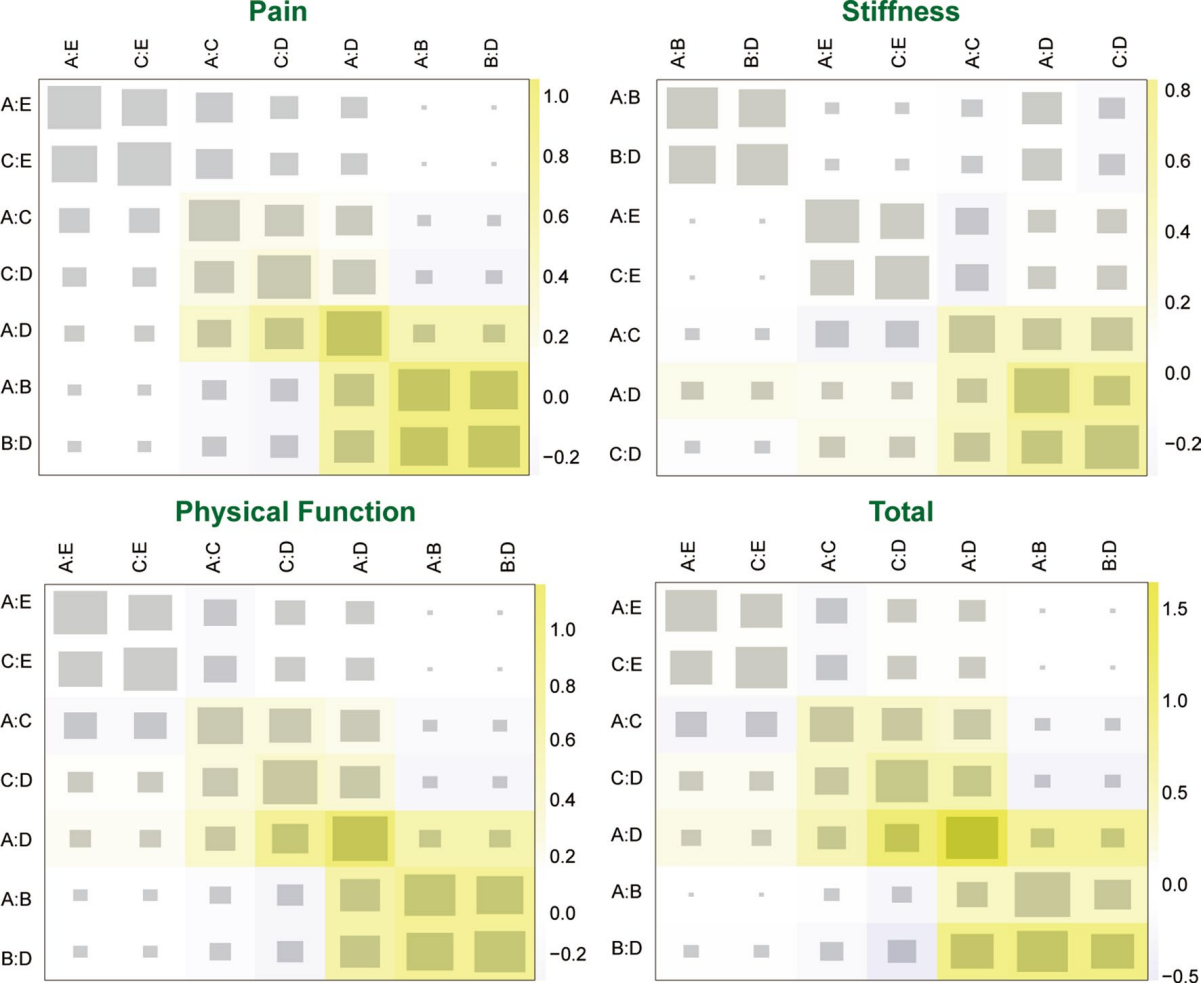
Heat plots of four outcomes: pain, stiffness, physical function and total (sum of the other 3 outcomes). *A* hyaluronic acid, *B* triamcinolone, *C* platelet-rich plasma, *D* saline, *E* hyaluronic acid + platelet-rich plasma

**Fig. 5:**
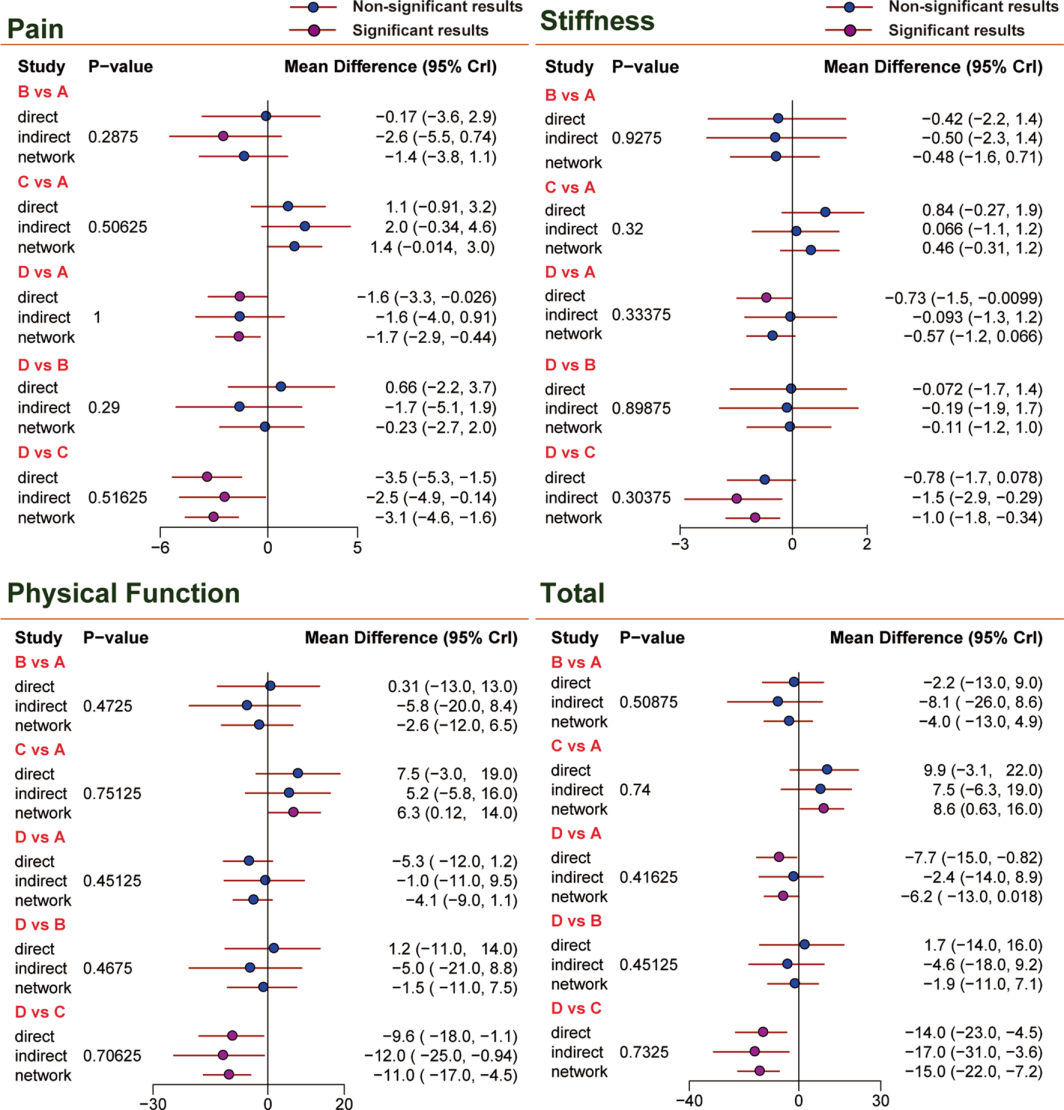
Node-splitting plots of four outcomes: Pain, Stiffness, Physical Function and Total (sum of the other 3 outcomes). 95% mean difference credible intervals (CrIs) indicates relative efficacy of treatment compared to another treatment. *A* hyaluronic acid, *B* triamcinolone, *C* platelet-rich plasma, *D* saline, *E* hyaluronic acid + platelet-rich plasma

**Fig. 6:**
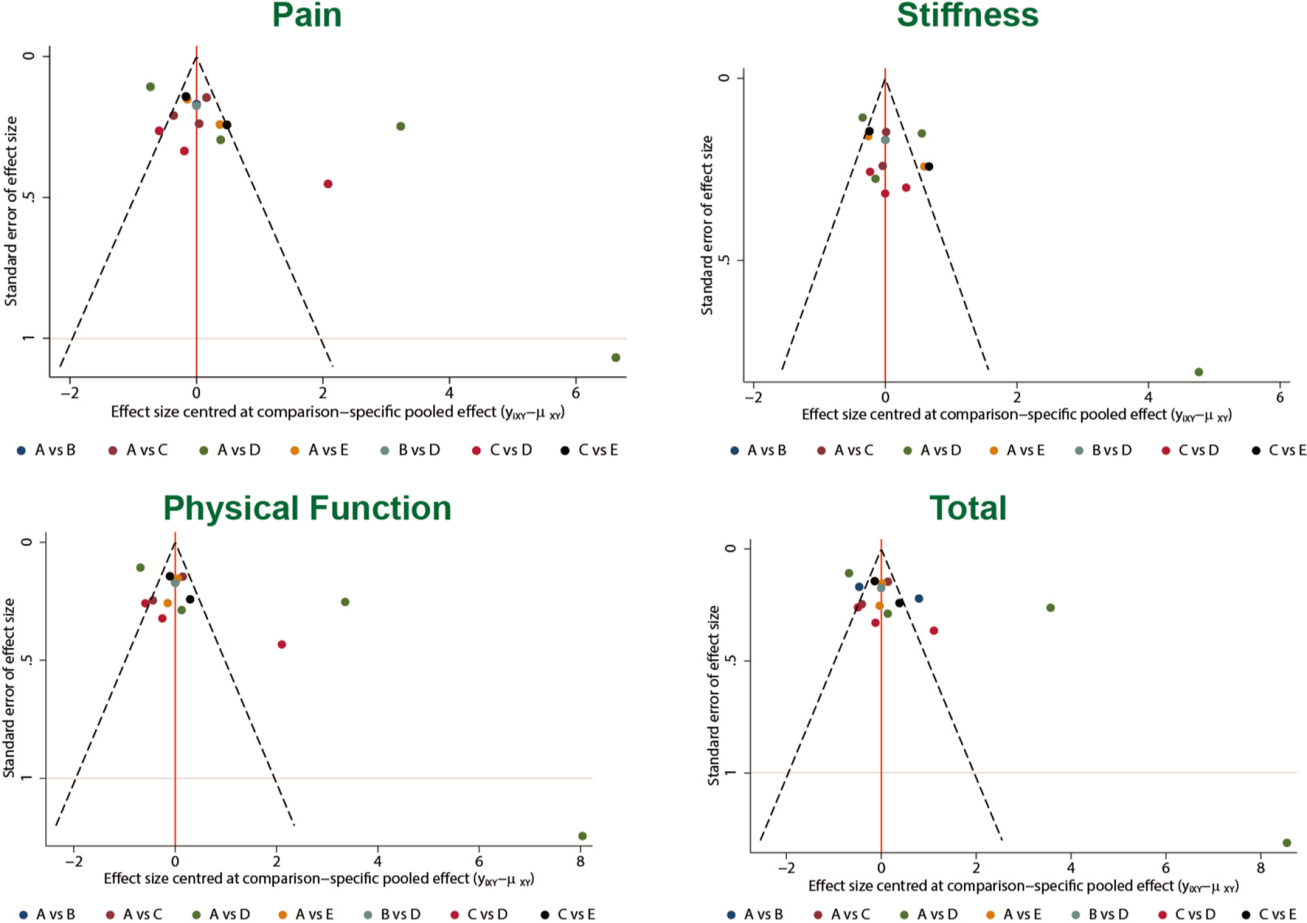
Comparison-adjusted funnel plots of treatments of 4 outcomes’ networks. The red line represents the null hypothesis that the study-specific effect sizes do not differ from the respective comparison-specific pooled effect estimates. *A* hyaluronic acid, *B* triamcinolone, *C* platelet-rich plasma, *D* saline, *E* hyaluronic acid + platelet-rich plasma

There was no evidence of inconsistency between direct and indirect estimates in the comparison among different studies, as seen in the heat plot and the node-splitting plot (Figs. 4 and 5). No evidence of publication bias is observed in Fig. 6.

## Discussion

Knee OA is a chronic progressive joint disease and the second main cause of disability, followed by a heavy social and economic burden [30]. Since the optimal treatment for knee OA patients remains inconclusive, the present NMA is performed to determine the best regime for patients with knee OA and provide a medical guideline. In the present study, the most important finding in this study is that HA + PRP showed the best efficacy in relieving stiffness, improving physical function and total scores, while PRP took the first place in terms of pain reduction.

As for stiffness, physical function and total scores, HA combined with PRP had the highest SUCRA value of 0.743, 0.772 and 0.765, respectively, which means that according to these three appraisal tools, HA combined with PRP is the first choice. This result was in accordance with the conclusions of some previous studies. The results of Yu et al. indicated that patients with knee OA who were treated with the combination of HA and PRP had more positive effects on body pain, and reduced arthralgia, bone damage and cartilage destruction, when compared with HA or PRP treatment alone [31]. Lana’s et al. study showed that HA and PRP combination treatment enjoyed better outcomes than HA alone up to 1 year and PRP alone up to 3 months [17]. Therefore, HA + PRP is the preferred therapy for knee OA patients.

Meanwhile, based on the SUCRA results of this NMA, PRP was another effective regime, as it ranked the first in pain reduction (0.691) and the second in stiffness (0.603), physical function (0.608), and total scores (0.624). A RCT study found that compared with therapeutic exercise alone, PRP injections and therapeutic exercise combination treatment could be more effective in reducing pain and improving stiffness as well as quality of life [32]. Another prospective, double-blind RCT supported that PRP injections had a better short-term effectiveness than placebos in the reduction of pain and stiffness and the improvement of knee function in early knee OA [33].

In the current study, we can see that irrespective of whether the HA was combined with PRP injection or the PRP injection alone, the key factor of these two treatments is PRP. Researches have indicated that inflammatory cytokines serve a critical function in the induction and development of OA [34, 35]. PRP is an autologous and multifunctional platelet concentrate of the blood that contains highly concentrated platelets and highly levels of cell growth factors. PRP promotes synovial cell proliferation and differentiation, as well as recovery of cartilage morphology [31]. That may be the key reason why the injection containing PRP shows an ideal outcome. Although plenty of studies have proven the efficacy of PRP in treatment of OA clinically, more mechanism research about OA and interaction research about the relationship between OA and PRP is required.

Although this NMA addressed many controversies on the clinical efficacy and safety of different treatments, there were still some limitations of the present study that require to be acknowledged. The limitations of our study include the following: (i) English studies were included, while non-English language articles without English abstract were omitted, which may lead to language bias; (ii) the study used self-reported questionnaire named WOMAC to evaluate outcomes, including pain, stiffness, physical function and total scores, which might limit the objectivity of results; (iii) due to insufficient data, the subgroup analysis in terms of severity of the disease, the frequency and dosage of the medication and the degree of activity has not been performed in this NMA. The fact of paucity of homogeneous studies also indicated to us that more high-quality and homogeneous studies should be carried out to provide us more strong evidence of this topic; (iv) the study sample size was relatively small, ranging from 10 to 174, which might have a negative effect on the conclusions. Hence, further high-quality RCTS are needed to address uncertainties regarding various treatments as well as qualify clinical efficacy.

## Conclusions

In conclusion, this NMA revealed that HA + PRP demonstrated the best efficacy in the improvement of stiffness, physical function, and total scores, while PRP had the best performance in terms of pain reduction. Ultimately, we hope that more and more researchers to compare the efficacy and safety of different regimes for patients with knee OA, and thus there will be more data and literatures to be referenced, which will provide more theoretical basis for clinical therapy of knee OA.

## Data Availability

All data generated or analyzed during this study are included in this published article.

## Abbreviations

CrI: Credible interval
MD: Mean differences
NMA: Network meta-analysis
OA: Osteoarthritis
PRP: Platelet-rich plasma
RCTs: Randomized controlled trials
SUCRA: Surface under the cumulative ranking curve
WOMAC: Western Ontario and McMaster Universities Osteoarthritis Index
